# Regular matters: credibility determination and the institutional habitus in a Swiss asylum office

**DOI:** 10.1186/s40878-020-00215-z

**Published:** 2021-01-29

**Authors:** Laura Affolter

**Affiliations:** Institute of Social Anthropology, University of Bern, Lerchenweg 36, 3012 Bern, Switzerland

**Keywords:** Administrative work, Asylum, Bureaucracy, Credibility, Discretion, Practice theory, Institutional habitus, Organisational ethnography, Refugee status determination, Switzerland

## Abstract

This article seeks to understand a common and regular feature of asylum decision-making, namely, that the majority of asylum claims are rejected, mostly on the basis of non-credibility. It draws on a bottom-up, qualitative study of an administration in which asylum decision-making takes place: the Swiss Secretariat for Migration. By adopting a practice-theoretical approach to administrative work, it advocates paying attention to caseworkers’ routinised, self-evident and largely unquestioned behaviours, not only in terms of what they do, but also of what they think, feel and know. Building on Bourdieu, it introduces the concept of institutional habitus, which refers to the dispositions caseworkers develop on the job. On the basis of a specific decision-making practice termed ‘digging deep’, the article shows how these dispositions are structured and how, through the practices institutional habitus generates, these ‘structuring structures’ are continuously reaffirmed, leading to the relatively stable outcomes of administrative decision-making that can be observed from the outside. The article argues against the assumption that regularities of administrative work should be understood as the outcome of strict rule-following, top-down orders and political instrumentalism. At the same time, it challenges the individualist quality sometimes ascribed to discretionary practices in street-level bureaucracy literature and in critiques of credibility assessment practices in asylum adjudication.

## Introduction: Regularities of administrative practice

The first Asylum Act regulating the legal categorisation of so-called asylum seekers in Switzerland was enacted in 1981. I use the word ‘so-called’ here because the term asylum-seekers is one specific to the ‘individual protection regimes’ of the global North which consider people to be ‘individual “asylum seekers” whose “well-founded fear of persecution” has to be examined in a laborious administrative procedure before they may become “refugees” legally’ (Poertner 2018, p. 5). This stands in contrast to the collective protection regimes of the global South where ‘people are collectively regarded as “refugees” because they fled their countries of origin or residence’ (ibid.). Since the early 1980s, the number of people applying for asylum in Switzerland has both increased and fluctuated greatly, and there has been considerable variation as to which countries most of the asylum seekers have been fleeing from (see Parak 2020).^1^ Yet, there is a consistency. Recognition rates have remained low since 1983, when the rate dropped from 74.9% to 48.6%, and have varied since then between 3.2% in 1991 and 31.2% in 2019.^2^ Furthermore, a trend that has been observed not only for Switzerland but also for other countries of the global North is that the majority of asylum applicants are rejected not because their claims do not fulfil refugee status requirements, but rather because they are not perceived as being credible, which is a legal precondition for being recognised as a refugee (see Fassin 2013, p. 47; Kelly 2012, p. 759; Tomkinson 2018, p. 186). This article seeks to understand such regularities in and of administrative practice through a bottom-up, qualitative study of the organisation in which administrative decision-making happens. It discusses and analyses the everyday practices of caseworkers^3^ in the Swiss Secretariat for Migration (SEM), which is the state administration in charge of taking first instance asylum decisions in Switzerland.

According to Max Weber’s ideal type of bureaucracy, stability and ‘calculability of results’ are key characteristics of bureaucracy and of legal-rational authority (Weber 2013 [1978], p. 223).^4^ Weber’s abstract theorisation of bureaucracy suggests that such stability is, at least in part, the result of rule-bound conduct (Weber 2013 [1978], p. 218). Yet, as Weber’s concept of the ideal type itself implies, in practice, administrative work functions differently, consisting of more than mechanical rule-following (see Lipsky 2010; Maynard-Moody and Musheno 2003). Not only is not all administrative practice guided by rules. Rules or law, whose ‘application’ lies at the heart of administrative decision-making, must necessarily always be interpreted when being fitted with specific situations or cases (see Eckert 2020, pp. 15–16; Eule et al. 2019, p. 101; Wagenaar 2004, p. 651). Hendrik Wagenaar calls these acts of interpreting rules and putting them into practice discretion and thus gives the term a meaning that differs from how it is commonly used in ‘classical’ street-level bureaucracy literature (Wagenaar 2020 p. 261). Michael Lipsky (2010, pp. 13–14) and Tony Evans (2010, p. 2), for example, conceptualise discretion in terms of the power and individual freedom so-called street-level bureaucrats have to make judgements and the way they selectively invoke different rules or law when dealing with specific cases. Discretion is depicted as the outcome of caseworkers’ relative freedom from supervision, allowing them to take actions or decisions – within certain boundaries – that they personally see as fit. Whilst these authors also recognise the necessity of discretion for making law and policy work, they ascribe an individualist quality to what they perceive as this discretionary freedom (see Eckert 2020, pp. 10, 15–16). Hence, little or no attention is paid to how caseworkers’ views of what they personally regard as fit, adequate, correct or appropriate are shaped by their office, nor to how certain interpretations of the law become normalised, and thus become the self-evident thing to do. This is, in contrast, precisely where my interest lies, for which I draw on Hendrik Wagenaar’s conceptualisation of discretion.^5^

Wagenaar claims that ‘[m]ost administrators in a particular policy domain or government bureaucracy operate in broadly similar ways’ (Wagenaar 2020, p. 260). He argues that while such similarities can ‘partly [be] explained by the behavioural shaping by organizational routines, standard operating procedures and software architecture, [ …] [these] do not explain how the discretionary part of all these decentred administrative actions is shaped’ (ibid.). This article takes up Wagenaar’s point, asking how caseworkers’ everyday interpretations of rules are shaped and structured by the organisational, ideological and political environments the caseworkers work in, the practicalities of the job, as well as the knowledge they acquire through their experience in the service, but also how their everyday practices of interpreting and ‘applying’ rules are constitutive of those very environments. It does so through a practice-theoretical approach to administrative work.

In recent years, several studies have addressed the issue of asylum decision-making from a bottom-up perspective, studying different asylum administrations and courts from the inside and providing insights into the everyday making of asylum decisions (see Dahlvik 2018; Johannesson 2017; Jubany 2017; Liodden 2016; Miaz 2017; Poertner 2018; Probst 2012; Scheffer 2001; Tomkinson 2018; and the edited volumes by Gill and Good 2019 as well as Lahusen and Schneider 2017). With a few exceptions that compare different national asylum administrations with each other (see Hamlin 2014; Probst 2012), the empirical focus of these studies lies on single national administrations (or courts) and their work. Read together, however, these ethnographic case studies bring to light striking resemblances in how caseworkers go about doing their job, despite differences in local contexts and organisational structures. In more or less explicit ways, the above-mentioned studies – as well as those of scholars working on administrations more generally – are concerned with the shaping of these everyday practices. Together they bring numerous influencing factors to light: induction training and organisational socialisation; professional norms, values and role perceptions; internal regulations and guidelines; structural constraints of ‘case-making’ and ‘fact-finding’; pragmatic considerations; commitment towards ‘clients’; accountability towards peers, intra- or supra-organisational authorities and the general public; and the broader political environment in which the organisations are embedded (Alpes and Spire 2014; Dahlvik 2018; Eule et al. 2019; Fassin and Kobelinsky 2012; Heyman 2004; Johannesson 2017; Jubany 2017; Liodden 2016; Maynard-Moody and Musheno 2012; Miaz 2017; Poertner 2018; Scheffer 2001; Tomkinson 2018; Zacka 2017). Furthermore, several scholars have explored the impact caseworkers’ educational background, gender, professional experience and/or political ideology has on their decision-making (see Anker 1992; Fassin and Kobelinsky 2012; Macklin 2009; Spirig 2018). By drawing on and engaging with these studies, the argument this article makes is twofold. First, in line with several of the above-mentioned studies, I argue in favour of paying attention to the regularities in administrative work and to the different factors giving rise to them instead of focussing on the divergencies in asylum decision-making. Connected to this, I propose to focus more on the structuring and stabilising factors working from within organisations than on caseworkers’ individual attributes, in order to gain a critical understanding of asylum decision-making (see also Tomkinson 2018). Second, building on the work of Pierre Bourdieu, I develop the concept of institutional habitus as a conceptual tool for analysing the structuring of discretionary practices. I conceptualise institutional habitus as the dispositions to think, act and feel in specific ways that caseworkers develop on the job (see Affolter 2021).

As Hendrik Wagenaar argues, ‘organizational routines, standard operating procedures and software architecture’ are not enough to ‘explain how the discretionary part of [ …] administrative actions is shaped’ (Wagenaar 2020, p. 260). The concept of the institutional habitus helps to address this ‘problem’ in that it not only allows us to bring together the different structuring factors that scholars have identified, but also in that it draws attention to how everyday practices structure their environments. Or, in other words, practices are not only constituted by their environments, they are also constitutive of them. Furthermore, the institutional habitus acknowledges knowledge in its different forms – implicit (or tacit), explicit, motivational, bodily and emotional – as a crucial part of what practice and, therefore, administrative work is. Thus, as I show in this article, only by analysing what this knowledge consists of and how it is acquired, passed on and reproduced can we gain an understanding of how caseworkers’ discretionary practices – their everyday acts of interpreting law when fitting it with specific cases or situations – are shaped and structured.

The remaining sections of this article are structured as follows. I start by describing how administrative work can be conceptualised from a practice theoretical perspective and why this is useful for understanding asylum decision-making in the SEM and then introduce readers to the concept of the institutional habitus. The second part gives a short outline of how refugee status determination in Switzerland works and how I studied these processes in the SEM. It discusses the role of ‘talk’ when studying administrative work from a practice theoretical perspective. The third part analyses a specific practice which I call ‘digging deep’. It maps out structural factors that shape digging deep and shows how disbelief becomes institutionalised and reproduced through this very practice. In the conclusion, these findings are discussed against the backdrop of the institutional habitus.

## Asylum decision-making as practice and the institutional habitus

In conceptualising administrative work as practice, I draw on Andreas Reckwitz (2002, 2003), Hendrik Wagenaar (2004) and Julia Dahlvik (2018). A ‘practice’, according to Andreas Reckwitz, ‘is a routinized type of behaviour which consists of several elements, interconnected to one other: forms of bodily activities, forms of mental activities, ‘things’ and their use, a background knowledge in the form of understanding, know-how, states of emotion and motivational knowledge’ (Reckwitz (2002, p. 249). Understanding administrative decision-making as practice thus means recognising such everyday actions as an assemblage of all these different elements. Not only bodily activities matter – which in the case of administrative work mainly consist of activities such as writing, reading and talking. Different forms of knowledge, including implicit or tacit knowledge which refers to the things we simply know or can do without being able to say how and why, also constitute an important part of practice (see Dahlvik 2018, p. 57; Reckwitz 2003, p. 290).

The relevance of such implicit or tacit knowledge became immediately apparent when I observed how caseworkers do credibility assessments in the SEM and talked to them about the process. When I asked SEM caseworkers, in a rather abstract way, how they knew whether something was credible or not, they often referred to their ‘gut feeling’. For example, Andrea,^6^ a fairly experienced caseworker in the SEM, said to me: ‘It’s more like a feeling. And then I look for it in the text’. What she meant was that credibility assessment usually starts with an intuition about whether the asylum seeker’s story is true or not. This initial intuition – caseworkers’ implicit or tacit knowledge – then crucially guides their subsequent actions: how thoroughly they question applicants in the asylum interviews and what kind of arguments they look for in the minutes of the interview to reason their final written decisions.

Even if caseworkers themselves regularly referred to such ‘feelings’ or ‘intuitions’ as being ‘subjective’, I soon noted during fieldwork that caseworkers frequently concurred with each other in their impressions regarding the ‘authenticity’ of asylum claims, while for me – the outsider with no professional experience in the office – this was often much less clear. This impression was reaffirmed by my observation that caseworkers’ credibility assessments are rarely checked by their superiors. When asked about this, the superiors explained to me that this had to do with them knowing how their experienced employees worked and trusting them to be able to take the right credibility decisions. One superior, Nora, said to me:

All these terms [like ‘plausible’, ‘comprehensible’, ‘logical’ and ‘realistic’ for example] are used [for reasoning positive decisions]. For me that is ok. […] I mean, if someone uses a word like that who only started [working here] three months ago, I might ask: ‘Hey, what does that mean for you?’ But if [the decision] comes from someone whom I consider to be a valuable, serious, good employee, then I’ll allow it, because I know, I can imagine what it means for them.^7^

Nora’s statement indicates that she shares an understanding with her experienced employees of what it means for an asylum seeker’s story to be ‘realistic’, for example. For her, this means that there is no need to check the arguments of the written decision against the minutes of the asylum interview or to thoroughly study the minutes in order to evaluate how the interview was conducted. She trusts her experienced employees to do those things the right way. What makes Nora (and her fellow superiors) trust their experienced employees is that they have been sufficiently socialised by the office, which allows them to think and act in ways which are familiar to the heads and with which they agree. With new caseworkers, in turn, they see it as their duty to form the newcomers accordingly. Trust, therefore, depends on the institutional habitus caseworkers acquire.

This indicates that while habitus operates from within individuals, it is not strictly individual. Or, as Pierre Bourdieu argues, ‘[h]abitus is socialized subjectivity’ (Bourdieu and Wacquant 1992, p. 126). Conceptualising decision-making practices as ‘socialized subjectivity’ thus allows us to overcome the individualistic quality often associated with tacit knowledge, implicit know-how and personal feelings. This kind of ‘individualist bent’ (Eckert 2020, pp. 9–10) can be found in ‘classical’ street-level bureaucracy literature, as mentioned above, as well as in several critical studies of credibility determination in asylum procedures – conducted mainly by legal scholars – which criticise the so-called ‘subjectivity’ or even ‘arbitrariness’ of credibility assessments (see, for instance, Kagan 2003; Macklin 1998; Ramji-Nogales et al. 2009). Michael Kagan, for example, claims that ‘subjective assessments are highly personal to the caseworker, dependent on personal judgement, perceptions and dispositions, and often lacking an articulated logic’ (Kagan 2003, p. 374). His critique is that these assessments are ‘very difficult to review and are likely to be inconsistent from one decision-maker to another’ (ibid.). While I agree with him that such assessments are ‘personal judgements’ which are based on caseworkers’ dispositions, the point I make is that we cannot leave it at that, but must explore what these dispositions consist of, how they are acquired and how, through the practices they generate, they reproduce the conditions of their own existence. Hence, in the end these dispositions are less ‘personal’ than they may seem. I also do not challenge his assertion that there are inconsistencies (or, rather, divergencies) in decision-making. Research has shown that the outcomes of decision-making may differ between individual caseworkers (or judges) as well as different organisational units and administrations (see Anker 1992; Eule 2014; Fassin and Kobelinsky 2012; Hamlin 2014; Ramji-Nogales et al. 2009; Spirig 2018). Yet, I argue that these divergences should not be overestimated and that we must be careful not to lose sight of the regularities and commonalities that nonetheless exist, for instance, the fact that the majority of asylum applications are rejected on the grounds of non-credibility, even by those caseworkers considered the most ‘lenient’. Rejecting the assumption that administrative practices are highly individualistic does not mean that habitus works in a deterministic way (Hitchings 2012, p. 62). To the contrary, habitus is creative and inventive and can produce ‘an infinite number of practices’ (Bourdieu 1990, p. 55). However, the practices a habitus generates will necessarily fall within ‘the limits of its structures’ (Wacquant 1992, p. 19) and thus produce the relatively stable outcomes of administrative work that can be observed from the outside.

For Bourdieu, the dispositions which constitute a habitus are shared by people belonging to the same social class or social collective. With the institutional habitus, in turn, the focus is on the dispositions that emerge from and through organisational socialisation, the experiences members of an organisation make on the job, and their daily interactions with each other and their ‘clients’, rather than on caseworkers’ social background. This is not to say that their background does not have an influence on their everyday practices. Instead, the argument I make is that caseworkers’ social backgrounds alone are not enough to explain what they think, feel, know and do, and, thus, ultimately, to understand the work of organisations. To gain such an understanding, we must study the professional values, routines and knowledge that caseworkers pick up and come to incorporate into their work through their socialisation within the organisation. At the same time, the similarities different bottom-up studies of asylum administrations bring to light suggest that legal-administrative decision-making and refugee status determination as forms of ‘social institutions’ – understood in an abstract sense as a complex of norms, values, rules, roles, conventions and cultural-cognitive schemes (see Lang, Pott and Shinozaki, this volume; Miller 2019) – are constitutive of a habitus that can be observed not only in individual organisations. This is why I propose the term institutional habitus instead of organisational habitus.^8^ Nevertheless, specific organisations as field sites matter: they are the places in which such institutions converge and in which actors negotiate their environments (Wagenaar 2004, p. 648). Organisational structures, such as the ways in which induction training is organised or internal chains of accountability, shape caseworkers’ institutional habitus.

## Studying exclusion and inclusion from within the organisation

‘There is no bureaucracy without classification, without the invention of categories of inclusion and exclusion’, Don Handelman writes (1995, p. 280). This also applies to the SEM and asylum administrations more generally. In the SEM, caseworkers must assign asylum seekers to one of four legal categories: refugee with asylum, refugee with temporary admission, non-refugee with temporary admission and non-refugee without temporary admission. For making this categorisation, the two questions decision-makers must answer are: Are the applicant’s claims credible, and are the criteria for refugee status fulfilled? Refugees, according to Article 3 of the Swiss Asylum Act (AsylA),^9^ are defined as ‘persons who in their native country or in their country of last residence are subject to serious disadvantages or have a well-founded fear of being exposed to such disadvantages for reasons of race, religion, nationality, membership of a particular social group or due to their political opinion’ (Art. 3, paragraph 1, AsylA). In order to be recognised as a refugee, asylum seekers have to ‘prove or at least credibly demonstrate their refugee status’ (Art. 7, paragraph 1, AsylA). Article 7 of the Asylum Act further stipulates that one’s ‘[r]efugee status is credibly demonstrated if the authority regards it as proven on the balance of probabilities [and that] [c]ases are not credible in particular if they are unfounded in essential points or are inherently contradictory, do not correspond to the facts or are substantially based on forged or falsified evidence’ (Art. 7, paragraphs 2 and 3, AsylA). In practice, since material evidence for corroborating reasons for having fled often does not exist, credibility assessments are largely based on the applicants’ statements as they were recorded in writing during the asylum interviews. It is then up to the decision-makers to interpret for each specific case what it means for the narrative to be sufficiently founded or inherently contradictory, for example. Normally, asylum seekers are interviewed twice in the course of the proceedings. The caseworker who carries out the second, longer asylum interview usually takes the decision.^10^

Studies that research ‘practice’ have generated considerable debate as to whether observing ‘talk’ yields an adequate basis for grasping ‘practice’. Some authors have argued that it is not, since many of the things we do are partially non-linguistic (see Halkier and Jensen 2011; Martens 2012). At the same time, authors like Russel Hitchings (2012) hold that we should not underestimate the things that can be verbalised and what we can learn from talking to our interaction partners. As human beings, we are able to reflect on the things we do and to some extent also on the things that make us do what we do. Therefore, talking to people during fieldwork can be useful for learning about such reflections. Drawing on both these positions, I made use of method triangulation, combining participant observation with interviews and document analysis. Beyond providing us with insights into caseworkers’ own reflections, I find ‘talk’ useful in that it points us to the limits of what can be verbalised, indicating the moments in which implicit knowledge comes to bear. The following statement made by a SEM caseworker illustrates this:

Well, I have to say, in interviews you realise quickly if it’s credible or not, if a person has experienced something or not. Um, it’s difficult to put into words. […] [T]he narrative is just different from a person’s who is lying.^11^

In addition, as Steven Maynard-Moody and Musheno have argued, ‘talk’ in the form of story narratives is useful for gaining an understanding of the ‘normative reasoning and context’ that shapes administrative work, since ‘[t]hrough narratives, storytellers reveal more than they consciously know’ (Maynard-Moody and Musheno 2012, p. 21). Consequently, during fieldwork I made sure to always ask my interaction partners for specific examples. Often, however, this was not even necessary, with stories automatically finding their way into our conversations.

I conducted fieldwork in the SEM during various stays between 2013 and 2015. A major part of this field research consisted of accompanying caseworkers from four different asylum divisions in their daily work, observing and asking them about what they were doing, listening to their explanations and occasionally also actively joining in their activities (see McDonald 2005; Mugler 2019, p. 54). I attended asylum interviews, prepared these interviews with the caseworkers and discussed ‘cases’^12^ with them before and after the interviews. Furthermore, by sitting in their offices, I observed caseworkers carry out tasks such as writing decisions or giving advice to co-workers. A few times, I was also able to accompany the heads of asylum divisions during their daily routines. On those days, I observed superiors assign new asylum ‘cases’ to their employees and check the latter’s decisions, and I was able to accompany them to different meetings. Furthermore, many people kept stopping by the heads’ offices to discuss specific issues, allowing me to observe their interactions. Particularly fruitful moments of fieldwork were also the morning coffee breaks that the staff of an asylum division tended to take together. Finally, I was able to take part in the three-week training course for new asylum caseworkers in the SEM. All these moments of (participant) observations were complemented by interviews which I conducted with twenty-seven caseworkers from nine different asylum divisions and document analysis, mainly of induction training materials, different documents given to me by caseworkers and of seventy-two case files.

## ‘Digging deep’: regularised disbelief and its everyday (re-)production

The fact that most asylum claims end up being rejected on the basis of non-credibility has to do with a routinised practice which I call ‘digging deep’ (see Affolter 2020, 2021). Digging deep refers to the practice whereby decision-makers interrogate asylum seekers during asylum interviews in a way that is aimed at ‘discovering’ indicators of so-called non-credibility. Hence, decision-makers ask as many questions in asylum interviews and undertake as many extra investigations as they feel are needed until they have enough arguments for rejecting a claim or they are convinced that a story is true ‘after all’. This practice is not only characteristic of the SEM, but has also been described for other asylum administrations (see Bohmer and Shuman 2008, p. 136; Jubany 2017, pp. 137, 135; Kelly 2012, p. 765). Caseworkers themselves ‘merely’ call this practice testing credibility. What is crucial about this practice is that, as Thomas Scheffer’s (2001) detailed analysis shows, through the questioning techniques used in digging deep, the so-called ‘lies’, ‘liars’ and elements of non-credibility are not only passively discovered, but are actively generated. The following example from my fieldwork illustrates this.

The ‘case’ concerns an asylum applicant from Eritrea who is being interviewed by a caseworker called Bernard. As is common, Bernard, started the part about the applicant’s reasons for applying for asylum with an open question: ‘Ok, so now you can freely tell me about what happened to you personally in Eritrea and why you left Eritrea’.^13^ In his narrative, the asylum seeker recounted the following event: ‘Two or three armed people came to my home. They asked me questions. They insulted me verbally. Then they beat me. I don’t remember what happened after that [ … ]. They left us alone for roughly 20 days to a month. [ …] The soldiers then searched our house’.^14^ After that, Bernard began asking the asylum seeker ‘yes or no’ and ‘wh- questions’^15^:

Q: To confirm, just a quick question: If I’ve understood you correctly, the authorities came to your house twice. The first time you were interrogated and beat and the second time your house was searched. Is that correct?

A: Yes. They knew I had contact with [person A]. The government has spies everywhere.

Q: How much time passed in-between these two events, roughly?

A: A short time; about two or three days. They really wanted to know what [person A] had said to me.

Q: Can you name the date your house was searched and you left [your home town]?^16^

At the end of the interview, Bernard confronted the applicant with contradictions. This again is standard and also a formal administrative requirement, since it counts as granting applicants the right to be heard (*rechtliches Gehör gewähren*).

Q: You said today that you were left alone for about 20 days after the first interrogation by the authorities. Later you said that two or three days passed between the two interrogations. Which one is true?

A: It was two or three days. If last time I said 20 days I was referring to the time span before I left [my home town]. There were so many problems. That’s why I can’t remember everything too clearly.^17^

The example shows how through his questioning technique, Bernard created a possibility for comparison, in this case between the statements made by the applicant at different moments during the asylum interview. This is a typical component of digging deep. By asking the same question at multiple times during the asylum procedure or even during a single interview, caseworkers seek to draw comparisons between asylum seekers’ recorded statements from the first asylum interview and those from the second interview, between different statements from one and the same interview or between statements made by spouses or siblings who are questioned separately (see also Dahlvik 2018, p. 144; Scheffer 2001, pp. 160–165). In this case, by doing so, Bernard succeeded in creating a contradiction that – through linking it with the non-credibility criterion ‘inherent contradictions’ from article 7 of the Swiss Asylum Act – he used as a reason to reject the asylum claim. In his decision, he wrote:

Whilst in your free narrative you said that about 20 days to a month passed between the first time the authorities came to your house and the time the military searched your house (see file A9, p. 8), you later said that only two to three days had passed between the questioning by the authorities and the searching of the house (see file A9, p. 22).^18^

Bernard, therefore, used the applicant’s recorded statements from the minutes of the interview as an ‘on-file fact’ to reason and legitimise his decision.

Apart from searching for contradictions in this way, another strategy of digging deep is to test the so-called ‘substance’ of asylum narratives. What this means is that caseworkers try to find out whether asylum seekers can speak in a detailed manner about ‘events’ relevant for asylum, which is regarded as an important indicator of credibility. They do so by requesting things such as: ‘Please tell me in detail about the daily routine in prison’.^19^ Furthermore, caseworkers try to generate answers that they can then compare with ‘facts’ they can look up, for instance, by asking questions like ‘What was the name of your church that was bombed?’^20^ Many caseworkers told me that while interviewing asylum seekers, they always had the written decision in mind and kept questioning the applicant until they either had enough arguments to reject the case or were convinced that the story was credible. In recognising credible accounts, caseworkers (implicit) professional knowledge plays an essential role. Hence, caseworkers described ‘credible accounts’ to me as those which ‘just came pouring out’. All one therefore had to do was to ‘lean back and listen to them talk’.^21^ This (implicit) professional knowledge guides whether and how deeply caseworkers will dig in individual ‘cases’ and may also set the bar for specific groups of asylum seekers to fulfil this particular credibility requirement differently.

Digging deep was not something I only observed experienced caseworkers do. Newcomers do it too, albeit in a more prepared way with the help of lists they have created beforehand on the basis of the documents from the asylum seeker’s casefile. Therefore, the questions we must ask are, on the one hand, how digging deep becomes a professional necessity, making newcomers more or less do it right from the start, and, on the other, how it becomes a normalised, routinised practice.

In the three-week training modules for new asylum caseworkers, the trainees were repeatedly told that it was their duty to thoroughly test the credibility of asylum claims. They were taught the three-step questioning strategy that Bernard used for doing so. The idea is to start with open questions, then go on to ask ‘wh-questions’ and, finally, to ask ‘yes or no questions’ if necessary. The open questions at the beginning are intended to give asylum seekers the opportunity to tell their stories. At the same time, several caseworkers told me that this strategy of starting with an open question was useful because in their ‘free narrative’ (*freien Erzählung*) asylum seekers tended to get tangled up in contradictions (see also Jubany 2017, p. 136; Poertner 2018, pp. 161–162). In terms of credibility testing, the ‘wh-questions’ then serve the purpose of creating possibilities for comparison and testing ‘substance’, whilst the ‘yes or no questions’ aim to establish clear-cut ‘facts’.

Novices not only learn this questioning strategy in theory; they also practice it with their individual coaches who are usually experienced caseworkers working in the same organisational unit. Teresa’s statement when talking about her role as a coach, illustrates this:

New [officials] often don’t work with the criterion of ‘contradicts an inner logic’ [for reasoning credibility decisions]. To give you an example: In one case, an applicant said that his father had asked him to deliver letters to another person. And he didn’t just do that once, but more like four to five times a week for about half a year. Now the [new] decision-maker did not think to ask the applicant what was in those letters, so when we were preparing the interview together I told her she had to ask him that. When she did, the applicant said that he had never asked his father that out of respect. And that is just not logical. If I were your father and I gave you a letter to take to someone nearly every day, you would ask me, even if you respected [me]. You would say: ‘Of course I’ll take the letter, father, but what are those letters you’re asking me to deliver’. That’s the obvious thing to do. Now, say the applicant had asked his father and the father had said: ‘That’s none of your business’, that would have been relatively plausible. But that he didn’t even ask his father is just not logical. And that’s a strong argument [for the decision]. So that’s what I mean when I say that [new caseworkers] need to learn to ask good and suitable questions.^22^

The example shows how Teresa is teaching the novice to be sufficiently suspicious and alert towards indicators that there might be something ‘off’ about the story and how she is conveying a clear idea of what merits suspicion and what assumptions are deemed common sense when defining behaviour as ‘normal’ and ‘abnormal’ or ‘logical’ and ‘illogical’. The novice is taught that this particular common-sense assumption (that it is illogical to not ask about the letters) will make for a strong argument, but that coming up with such arguments is dependent upon the questions she asks. She is taught to dig deep.

Upon joining the office, newcomers are generally attributed a naivety that they first have to grow out of in order to not believe asylum seekers ‘too readily’. Hence, akin to what has been described for other asylum administrations as well as administrations more generally where people apply for goods and make rights claims on the state (see Alpes and Spire 2014, p. 269; Borrelli et al.: States of suspicion: How institutionalised disbelief shapes migration control regimes, forthcoming; Lavanchy 2014), I observed that sufficient – but not overt – suspicion was considered an important professional norm in the SEM. This norm is, on the one hand, linked to administrative caseworker’s role as ‘gate-keepers’ (Lipsky 2010, p. 4) or ‘guardians of a restricted good’ (Heyman 2009, p. 381), which in the case of decision-making in the SEM is the right to reside in Switzerland. On the other hand, the specific political and ideological context in which asylum decision-making takes place, further fuels this professional norm.

Since the 1980s, Swiss asylum policies have become increasingly restrictive (see Miaz 2017; Piguet 2006). Like in other countries of the global North, the ‘fight against abuse’ is high on the political agenda, accompanied by a widespread discourse on so-called ‘bogus refugees’ (see Zimmermann 2011). Asylum seekers and migrants in general are often portrayed as a ‘problem’ in political discourse and media and are seen as a ‘threat’ to states’ economy, culture and identity (see Dahlvik 2018, p. 9; Gill and Good 2019, pp. 5–6; Miaz 2017, pp. 11–12). Today, asylum determination procedures in the global North are thus structured around a ‘politics of deterrence’ (Poertner 2017), with no country wanting to be more generous in granting asylum and subsidiary protection than others, in order to avoid creating a so-called ‘pull-effect’ (see Jubany 2017, p. 62; Liodden 2016, p. 219; Miaz 2017, pp. 15, 343; Poertner 2017, pp. 17, 283). Exploring the full complexity how asylum decision-making practice is shaped by this political and ideological context goes beyond the scope of this article. However, the point I want to make here is that, rather than this political influence working in ‘direct’ ways – in the sense that it plays out through explicit top-down orders or through caseworkers personally sharing this political opinion (many explicitly do not) –, it more indirectly shapes caseworkers’ understanding of what it means to work in the name of the state. In this sense, fulfilling Switzerland’s duties under international law and maintaining its self-ascribed image as a humanitarian country, on the one hand, and securing Switzerland’s ‘borders’ by restricting non-citizens’ access to rights and goods as well as protecting the Swiss asylum system from so-called ‘abuse’, on the other, become important institutional aims. In a seemingly paradox way, both these ‘aims’ translate into an exclusionary logic of asylum. Hence, as Didier Fassin and Carolina Kobelinsky have shown for the French asylum system, ‘[t] he less frequently [asylum] is granted, the more precious refugee status becomes’ (Fassin and Kobelinsky 2012, p. 464). It is through this lens that we must understand Gabriel when he explained to me that it was the caseworkers’ duty to meticulously examine the credibility of each case because otherwise ‘everybody could just receive asylum and that would be unfair to those who really deserve asylum, who really need protection’.^23^ In this way, exclusion becomes normalised.

Finally, the emotional burden asylum decision-making poses for caseworkers as well as pragmatic considerations also encourage digging deep. What I mean by this is that it is often easier for caseworkers to reason negative decisions on the basis of non-credibility than on the basis of non-eligibility for refugee status, because they feel more secure about such decisions. Samuel’s statement exemplifies this:

Testing credibility is especially important if they [the asylum seekers] come from states that act in an arbitrary manner [*willkürliche Staaten*]. […] Or broadly speaking, the worse the situation in a country, the more we have to focus on the credibility of the claim, or the more we tend to argue on the basis of credibility. I guess we have to. Because in such countries even a minor political activity can quickly result in the person being persecuted.^24^

Samuel insinuates that, as an asylum caseworker, one often has no way of knowing whether an applicant might be in danger of future persecution in their ‘country of origin’ or not –in some countries, anything could potentially lead to a person being persecuted. However, the practices described in this article are perceived by caseworkers as a way of ascertaining with sufficient accuracy whether an asylum seeker’s claims are credible or not. Similarly, Patricia told me that it sometimes felt very cynical to her to say to someone, for example: ‘You have no reason to be afraid’ of going back to Syria. She felt, in contrast, that pointing out the contradictions in asylum seekers’ statements and raising doubts about an entire story was easier.^25^ Rejecting asylum seekers on the basis of non-credibility is, therefore, not only easier because such decisions can be reasoned with more certainty. By rejecting claims on the basis of non-credibility, the emotional burden of taking such life-changing decisions is lessened. The responsibility for the outcome of the decision is shifted to the asylum seekers; it is their fault for not telling ‘the truth’. Or as Helen said: ‘I can also tell you why [I prefer arguing negative decisions on the basis of non-credibility]. It’s for my conscience. If someone tells you rubbish [*dir einen Stuss erzählt*], then you don’t have such a guilty conscience’.^26^

## Conclusion

The aim of this article has been to address regularities in asylum decision-making using a bottom-up, organisational approach. Studying such regularities entails investigating the shaping and structuring of caseworkers’ interpretations of rules and law when ‘applying’ them to specific ‘cases’. At the core of this article were caseworkers’ interpretations of Article 7 from the Swiss Asylum Act or, in other words, how they assess the credibility of asylum claims. As I have shown, administrative practice can only be understood by taking into account the many different elements which constitute it: what caseworkers do (for example, the questioning techniques they use and the questions they ask); what they know (for instance, the gut feelings which guide their credibility assessment or their knowledge as to how to successfully reason decisions); and what they think and feel (for instance, the perceived necessity for and adequateness of digging deep or the emotional burden asylum decision-making poses for caseworkers). All this makes the institutional habitus a useful concept for grasping administrative practice in its complexity. Furthermore, the institutional habitus considers structuring factors, such as the formal requirements of legal-administrative decision-making as well as the political, organisational and ideological environments in which the latter takes place. But the institutional habitus is not only a system of ‘structured structures’; the practices it generates also work as ‘structuring structures’ (see Bourdieu 1990, p. 53). Digging deep is an example of this.

Through digging deep, caseworkers create the ‘on-file facts’ needed to reason negative decisions. In doing so, the figure of the ‘bogus refugee’ is continuously reproduced. Once asylum seekers have been classified as ‘bogus refugees’ by assigning them to the legal category of ‘non-refugee’, their existence becomes a fact, reinforcing the perception that, indeed, there ‘are’ many ‘bogus refugees’, which, in turn, intensifies the office’s and individual caseworkers’ efforts to identify and exclude them from asylum (see also Zimmermann 2011, p. 337). At the same time, if decision-makers reach the conclusion after digging deep (or due to their ‘gut feeling’) that there are no (or not enough) reasons for disbelieving an applicant, a certain image of the ‘genuine refugee’ is (re-)produced. Consequently, the practice of digging deep constructs the conditions that lie at its very basis.

Digging deep, in the particular way described in this article, seems to be specific to asylum administrations and decision-making. And some aspects may even be specific to the SEM, depending on the formal requirements for reasoning asylum decisions or on how caseworkers are trained within the organisation, for example. Moreover, although I did not systematically study these aspects, my research suggests that different organisational units within the SEM and also individual caseworkers differ with regard to how meticulously they dig deep. My interaction partners were prone to point this out to me during fieldwork, frequently criticising their fellow caseworkers (mostly from other organisational units) as being either too ‘soft’ or too ‘hard-line’ (see also Miaz 2017, pp. 374–375). However, unlike research which focuses precisely on such divergencies, the aim of this article has been to look beyond them – not to deny that they exist, but to shed light on the mundane ‘normalities’ of decision-making, in other words, on the unquestioned middle ground between different ‘extreme positions’. In-between ‘cynical’ or ‘hardline’ decision-making and ‘soft’, ‘lazy’ (in the sense of not digging deep enough) or ‘naïve’ decision-making, which are considered unprofessional, there are the largely unquestioned modes of decision-making that are marked by considerable suspicion and that are commonly accepted as professional. This does not mean that all caseworkers consciously consider themselves or even feel that they have to be suspicious. Rather, this middle ground manifests itself in the way in which digging deep is accepted as the self-evident and normal thing to do, even if the intensity of the process may be a matter of contestation. In other words, digging deep is considered as the epitome of professional, neutral and objective decision-making (see Affolter 2020).

By focusing exclusively on stabilising structures and practices, for the reasons outlined above, this article leaves its own blind spot, namely the transformative capacity that is also inherent in the institutional habitus. Researching how dispositions which structure everyday practice change over time and also how caseworkers, through their everyday practice, challenge certain structuring structures, should be at the core of future research.

Beyond asylum administrations, a practice-theoretical approach with its consideration of ‘the almost unthinking actions, tacit knowledge, fleeting interactions, practical judgements, self-evident understanding and background knowledge, shared meanings, and personal feelings’ (Wagenaar 2004, p. 643) would seem to be useful for investigating all settings of administrative work. Furthermore, thanks to its focus on structuring structures, the institutional habitus constitutes a useful tool for cross-organisational comparison, allowing researchers to distinguish organisation-specific influences from those that more broadly shape administrative practice beyond specific organisational settings.

## Data Availability

The data used for this manuscript is subject to privacy regulations and not available to the public.

## Footnotes

1 See also: SEM. Asylstatistik. Archiv ab 1994. https://www.sem.admin.ch/sem/de/home/publiservice/statistik/asylstatistik/archiv.html. Accessed 10 September 2020.

2 I thank Jonathan Miaz for sharing these numbers with me. The recognition rates refer only to the percentage of asylum seekers that were legally recognised as refugees and granted asylum. They do not include those people who received temporary protection on so-called humanitarian grounds.

3 I use the term caseworkers to describe officials working in administrative organisations because the people whose everyday practices I study assess and take decisions on individual claims. In dealing with these claims, they engage in case-making (see Poertner 2018).

4 The terms bureaucracy and bureaucrats carry negative connotations. They are often associated with red tape and officialism and used as criticism (see Eckert 2020, p. 7; Poertner 2017, p. 12; Zacka 2017, pp. 1–3). It is for this reasons that I employ the terms administration and caseworkers instead.

5 For a detailed discussion of the term discretion, see Affolter 2021), Eule et al. (2019, pp. 84–90) and Liodden (2016, pp. 68–76).

6 All the names I use in this article are pseudonyms.

7 Nora, head of asylum division, interview transcript, my own translation.

8 Similar concepts to that of the institutional habitus have also been used by Didier Fassin as well as Maybritt Jill Alpes and Alexis Spire. The term Didier Fassin briefly refers to in his introduction to *At the Heart of the State* is that of the professional habitus. He argues that officials’ ‘principles of justice [and] or order’ as well as ‘the values of the common good and public service’ are the products of officials’ professional habitus (Fassin 2015, p. 6). For Maybritt Jill Alpes and Alexis Spire, what they call the bureaucratic habitus ‘significantly shapes […] [caseworkers’] interpretations of the legal frameworks’ and consists of the ‘set of norms shared by other agents involved in migration control’ (Alpes and Spire 2014, p. 263).

9 Asylum Act (AsylA) of 26 June 1998 (Status as of 1 April 2020). https://www.admin.ch/opc/en/classified-compilation/19995092/index.html. Accessed 30 September 2020.

10 What I describe here are the procedures in Switzerland as they were applied before the introduction of the new accelerated asylum process on 1 March 2019. While this last reform has led to significant changes in the administration’s organisational structure and some procedural aspects have changed, the core of SEM caseworkers’ work has not. They still conduct asylum interviews and assess asylum seekers’ eligibility to refugee status as well as their credibility, which is what this article is about.

11 Ralph, caseworker, interview transcript, my own translation.

12 ‘Cases’ is an emic term. Of course, what SEM caseworkers really deal with are not cases but people whose lives are greatly affected by their practices and decisions.

13 Minutes of second asylum interview, field document, my own translation.

14 Minutes of second asylum interview, field document, my own translation.

15 ‘Wh- questions usually start with a word beginning with wh-, but ‘how’ is also included. The wh- words are: what, when, where, who, whom, which, whose, why, and how’ (Cambridge Dictionary. Wh-question. https://dictionary.cambridge.org/de/worterbuch/englisch/wh-question. Accessed 23 September 2020).

16 Minutes of second asylum interview, field document, my own translation.

17 Minutes of second asylum interview, field document, my own translation.

18 Asylum decision, field document, my own translation.

19 Minutes of second asylum interview, field document, my own translation.

20 Minutes of second asylum interview, field document, my own translation.

21 Daniel, caseworker, interview transcript, my own translation.

22 Teresa, caseworker, interview transcript, my own translation.

23 Gabriel, caseworker, interview transcript, my own translation.

24 Samuel, caseworker, interview transcript, my own translation.

25 Patricia, caseworker, interview transcript, my own translation.

26 Helen, caseworker, interview transcript, my own translation.
